# Safety and Tolerability of Overdosed Artificial Tears by Abraded Rabbit Corneas

**DOI:** 10.1089/jop.2018.0040

**Published:** 2018-12-06

**Authors:** Philippe Daull, Elisabeth Raymond, Laurence Feraille, Jean-Sébastien Garrigue

**Affiliations:** ^1^ SANTEN SAS, Novagali Innovation Center, Evry Cedex, France.; ^2^ Iris Pharma, La Gaude, France.

**Keywords:** cationic emulsions, cornea abrasion, *in vivo* model, wound healing, safety evaluation

## Abstract

***Purpose:*** Preservative-free cationic emulsion-based artificial tear (AT) is an innovative eye drop based on the Novasorb^®^ technology with cetalkonium chloride (CKC) as the cationic agent. The cationic emulsion Cationorm is designed for the management of mild-to-moderate dry eye disease (DED) patients that present cornea epithelium alterations. The aim of the present study was to evaluate the safety and tolerability of overdosed ATs by altered corneal epithelium *in vivo* and assess the usefulness of the *ex vivo* eye irritation test (EVEIT) as a predictive alternate toxicity test method.

***Methods:*** The experimental procedure, treatment duration, and instillation frequency closely mimic *in vivo* the *ex vivo* protocol described by Pinheiro et al. and discussed in the Discussion and Conclusion section of this article. Two to 3-month-old female New Zealand white rabbits, *n* = 6 per group, were treated with ATs (21 instillations/day over 3 days) following corneal abrasion. Corneal fluorescein staining, *in vivo* confocal microscopy (IVCM), and slit lamp examinations were performed to assess corneal epithelium recovery and the ocular tolerability of the overdosed ATs.

***Results:*** All abraded eyes experienced almost complete epithelium recovery within 3 days following treatments with Cationorm, Optive, Vismed, and Saline. Benzalkonium chloride (BAK, 0.02%) treatment resulted in 82.4% reepithelialization. IVCM data illustrated corneal epithelium normal recovery. Acute local tolerability of the overdosed ATs was confirmed using Draize and McDonald–Shadduck's test scales.

***Conclusions:*** The different ATs were demonstrated to be well tolerated by abraded corneas *in vivo*, and the extreme overdosing regimen did not hamper the wound healing process of the rabbit eye in comparison to saline. These data did not confirm the ones obtained with the nonvalidated *ex vivo* eye irritation test.

## Introduction

Dry eye disease (DED) is a complex multifactorial disease of the ocular surface that manifests by structural alterations of the cornea (keratitis) and the presence of symptoms of discomfort of variable severity (scratchy and dryness sensation, stinging or burning, pain, and eye redness).^1^ Patients with DED may also experience a blurred vision, eye fatigue, and heavy eyelid sensations. The tear film (TF), whose normal function is to protect, hydrate, and nurture the corneal cells and thus preserve vision's quality, is often altered and instable in DED patients, thus not able to play its roles.^2^ Artificial tears (ATs) represent the first line therapy for restoring the TF and for the management of the symptoms and signs of DED patients.^3–6^ Many different ATs exist with different formulation strategies used to restore a healthy TF.^7^ Aqueous-based AT formulations, with or without gel-forming polymers (eg, carboxymethyl cellulose, hydroxypropyl guar, and hyaluronic acid), are classically used in aqueous-deficient DED,^8^ while lipid-containing ATs are used for evaporative DED.^9–11^ For the temporary relief of the less severe (ie, mild to moderate) symptoms, patients are advised to instill one drop in the affected eye when needed and up to 4 drops per day.^12^ The demonstration of the efficacy and safety of most of the ATs was performed in 1–3 month clinical trials in mild-to-moderate DED patients treated with up to 4 instillations per eye and per day.^13–17^

The management of the more severe symptoms generally requests the need of prescription eye drop containing potent anti-inflammatory actives like cyclosporine,^18,19^ lifitegrast,^20,21^ or glucocorticoids.^22^ However, depending on the severity of the discomfort some patients might feel the need to instill ATs in their eyes above the recommended dosing regimen of 4 eye drops per day, with the risk of developing acute toxicity events. The safety of the ATs is generally demonstrated during the preclinical development stage of the ATs in *in vitro* and *in vivo* relevant animal models with dosing regimens exceeding the expected human posology. This approach usually allows for the definition of a margin of safety (for drugs under development), which can be defined as the range between the minimal therapeutic dose and the minimal toxic dose of a drug. However, it is often not possible to precisely determine a margin of safety for ATs, as for the latter the maximal dose assayed in the animal ocular tolerability/toxicology tests is not high/extreme enough for the development of adverse events to appear (and thus define the minimal toxic dose) due to the inherent very good safety of unpreserved ATs.

With the idea of reducing the use of animal models for toxicology and safety evaluations, alternative *in vitro* and *ex vivo* models are being developed. Unfortunately, most of them do not generate sufficiently reliable data to be validated and used as good alternative test methods.^23^ Recently, an *ex vivo* eye irritation test (EVEIT) was developed and used as an alternate assay model to evaluate the toxic effects of ATs on a wounded corneal epithelium subjected to hourly (24 instillations per day) instillations over a 3–6 day period. This highly overdosed dosage regimen in the nonphysiological experimental setting of the EVEIT assay suggests dramatic ocular toxicity of the preservative-free cationic emulsion Cationorm and the Purite^®^ preserved Optive eye drops. The goal of the present study was to evaluate the effects of these 2 eye drops in a rabbit model with similarly wounded corneas and receiving 21 instillations (one eye drop every 45 min) per day over a 3-day experimental period and, thus, assess the reliability of the EVEIT assay data.

## Methods

### Animals

Thirty female New Zealand white rabbits (Hypham, Roussay, France) of approximately 2–3 months of age were used in this study. Iris Pharma Internal Ethics Committee approved the protocol. All animals were treated according to the Directive 2010/63/UE European Convention for the Protection of Vertebrate Animals used for Experimental and Other Scientific Purposes and to the Association for Research in Vision and Ophthalmology (ARVO) statement for the Use of Animals in Ophthalmic and Vision Research. All animals were housed individually in standard cages under identical temperature (18°C ± 3°C), humidity (45%–80% relative humidity), and controlled enrichment conditions and exposed to a 12-h light/12-h dark cycle in continuously ventilated rooms (15 air volumes per hour). They received a standard dry pellet diet and water *ad libitum*.

### Experimental procedure and treatment groups

**FIG. 1.**
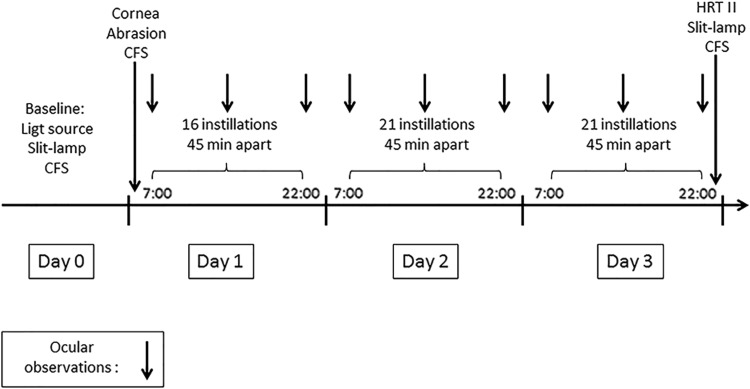
Schematic representation of the experimental design of the *in vivo* study.

The animals were randomly assigned to 5 groups (*n* = 6 per group) defined as follows: a negative control group receiving saline (0.9% NaCl), a toxic control group treated with a 0.02% benzalkonium chloride (BAK) solution (in saline), and 3 treatment groups: Cationorm (Santen SAS, Evry, France), Vismed (Horus Pharma, Saint-Laurent du Var, France), and Optive (Allergan, Inc., Dublin, Ireland). Twenty microliters of each eye drop was applied for up to 21 instillations (except on the day of induction with 16 instillations) per day (45 min apart) in the right eye and started 1 h after the completion of the corneal abrasion procedure. The treatment period lasted 3 days. The study design used in this experiment is presented in Fig. 1.

### Corneal abrasion procedure

**FIG. 2.**
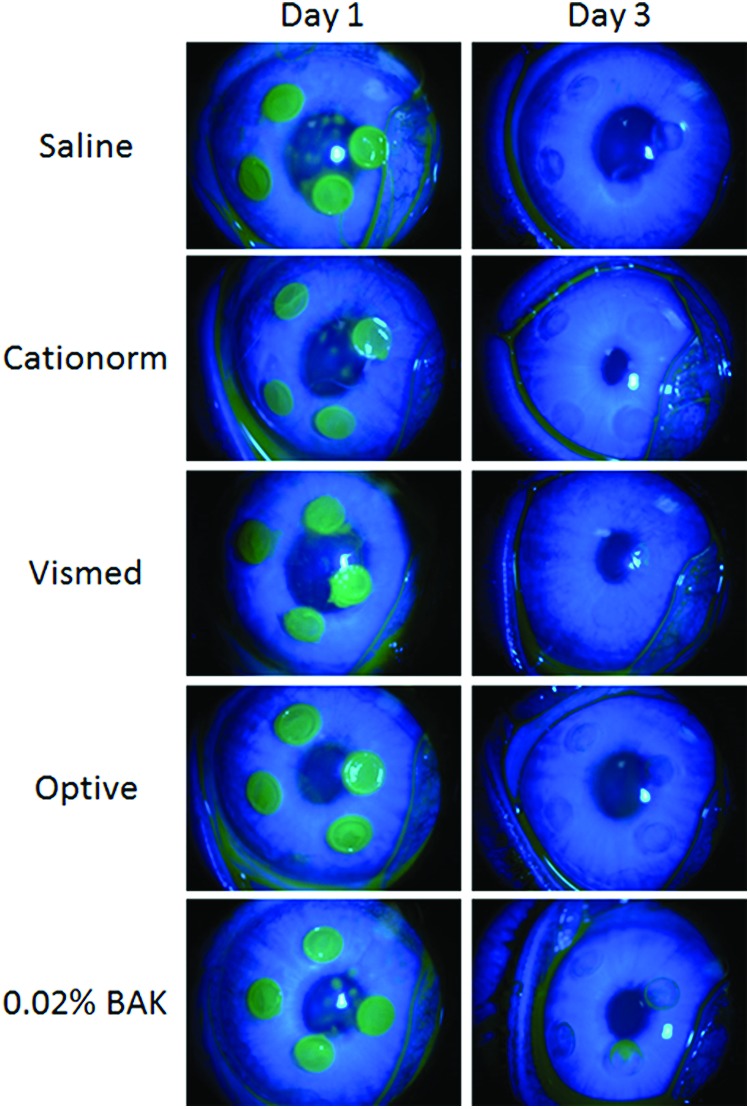
Illustration of the cornea defect during the experiment. Corneal fluorescein staining of abraded rabbit eyes at the beginning of the treatment period (day 1) and after 3 days of treatment (day 3). Color images available online at www.liebertpub.com/jop

Rabbits were deeply anesthetized by an intramuscular injection of a mixture of ketamine (35 mg/kg)/xylazine (5 mg/kg), and local cornea anesthesia was performed by instillation of a drop of 0.4% oxybuprocaine (Cebesine^®^, Laboratoire Chauvin, Montpellier, France) in the study eye. Standardized superficial corneal abrasions (4 per right eye) were performed with a classic biopsy punch (2 mm in diameter) to a depth of approximately 35 μm (Fig. 2). An analgesic (buprenorphine, 20 μg/kg, SC) was administered 30 min before corneal abrasion and once a day during the following 2 days.

### Corneal fluorescein staining and healing measurement

One drop of a 0.5% fluorescein sodium solution (Fluoresceine Faure, 0.4-mL unit-dose vials, Novartis Pharma SAS, France) was instilled into the inferior conjunctival sac using a micropipette. The cornea was examined through a biomicroscope by light passing through a cobalt blue filter. Pictures of cornea lesions (area stained by fluorescein) were taken using a CCD camera and analyzed using ImageJ software to measure the size of the 4 abrasions on each cornea.

### Local tolerability evaluation with Draize, McDonald–Shadduck, and IVCM scales

#### Ocular examinations for clinical evaluations

Both eyes were examined under a light source for cornea, conjunctiva, and iris adverse reaction scoring according to Draize's scale. Slit lamp examinations of both eyes were performed to assess cornea, conjunctiva, iris, and the inner parts of the eye adverse reactions according to McDonald–Shadduck's scale. Figure 1 presents the schedule of the different observations performed during the experiment; slit lamp observations were performed at baseline and at the end of the experiment, while ocular observations were also performed each day before the first and after the 10th and 21st instillation of the day.

### *In vivo* confocal microscopy

A laser scanning *in vivo* confocal microscope Heidelberg Retina Tomograph (HRT II)/Rostock Cornea Module (RCM, Heidelberg Engineering GmbH, Heidelberg, Germany) was used to examine the cellular structure of the cornea. Pictures (400 × 400 μm) of the superficial and basal epithelium, of the anterior and posterior stroma, and the endothelium were taken in the center of the cornea with at least one wounded area in the field of the microscope.

### Statistical analysis

Statistical analysis was performed with GraphPad Prism software, and intergroup comparisons were performed by 1-way or 2-way ANOVA followed by Tukey's multiple comparison test. The significance threshold was set at *P* < 0.05.

## Results

### Effects of overdosed ATs on the healing process

**FIG. 3.**
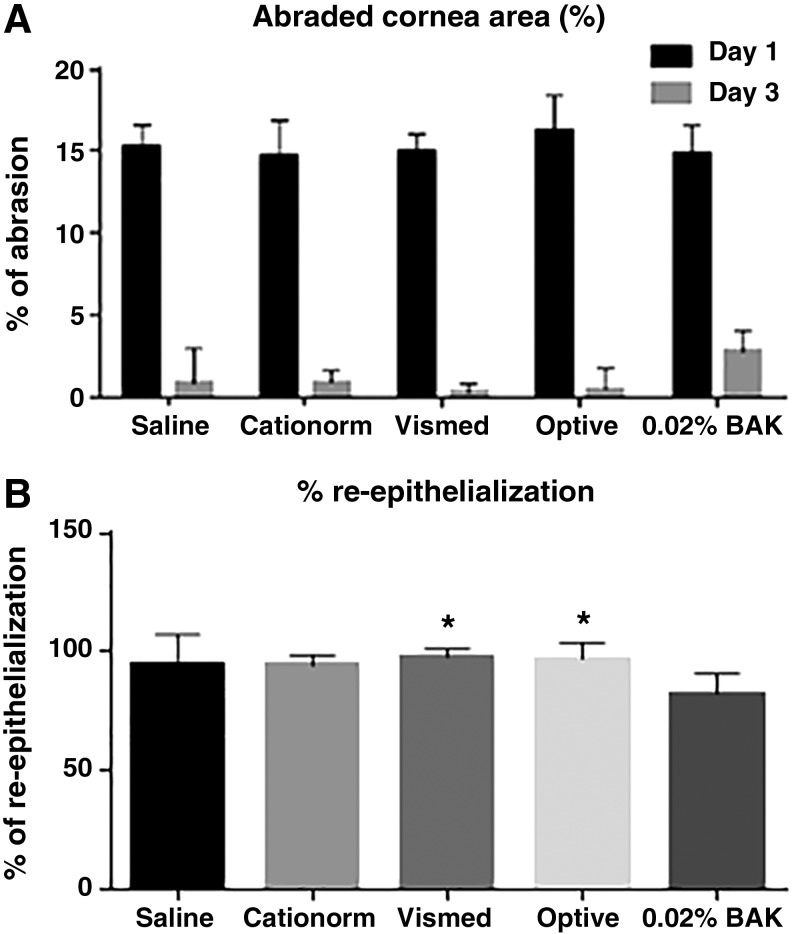
**(A)** Percentage of cornea abrasion (±SD) at day 1 and day 3 for the different treatment groups. There is no statistically significant difference between the groups (2-way ANOVA, Tukey's multiple comparison test). **(B)** Percentage of reepithelialization (±SD) at day 3. *P < 0.05 (1-way ANOVA, Tukey's multiple comparison test) versus 0.02% BAK.

Four standardized lesions were performed in the central part of the cornea (Fig. 2). The total abrasion area at day 1 (immediately after the debridement procedure) was approximately 15%, ranging from 14.8% ± 1.4% to 16.2% ± 0.5% for the different groups (Fig. 3A). Twenty one eye drops per day of various ATs were instilled for 3 days. At the end of the treatment period (day 3), the size of the remaining lesion was measured by fluorescein staining (Figs. 2 and 3B). The remaining lesion size varied from 0.3% ± 0.5% to 0.9% ± 2.1% for the 3 commercially available ATs and saline, with no statistically significant difference between them. Only the 0.02% BAK solution had a lesion at day 3 that remains visible with the naked eye (Fig. 2) following fluorescein staining. The area of the remaining lesion represents 2.8% ± 1.3% of the total cornea area. Corneal reepithelialization at day 3 was almost complete (from 94.4% ± 12.3% to 97.9% ± 3.5%) following the treatment with saline and the 3 commercially available ATs (Fig. 3B). Surprisingly, the 21 instillations per day of a 0.02% BAK solution were not accompanied by a worsening of the lesion, that is, an increase in the size of the abraded area; the healing process remained effective, even though it appeared to be slower.

### Ocular tolerability of overdosed ATs by diseased corneas

**FIG. 4.**
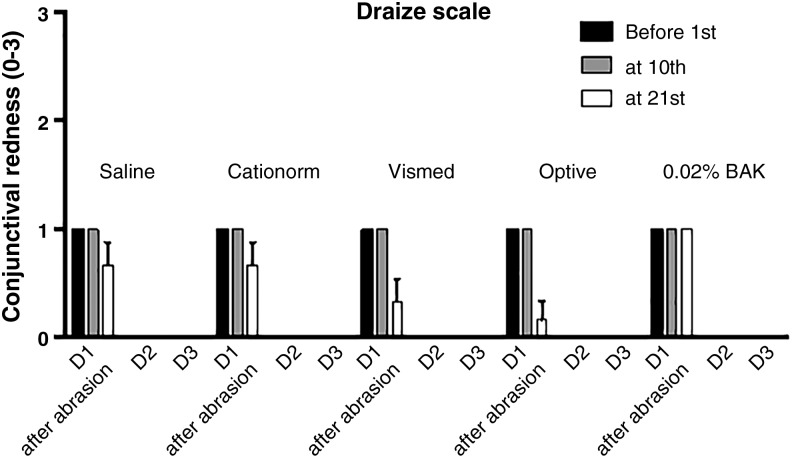
Mean Draize test score (±SEM) for the different treatment groups before the first and after the 10th and 21st instillations at days 1, 2, and 3.

**FIG. 5.**
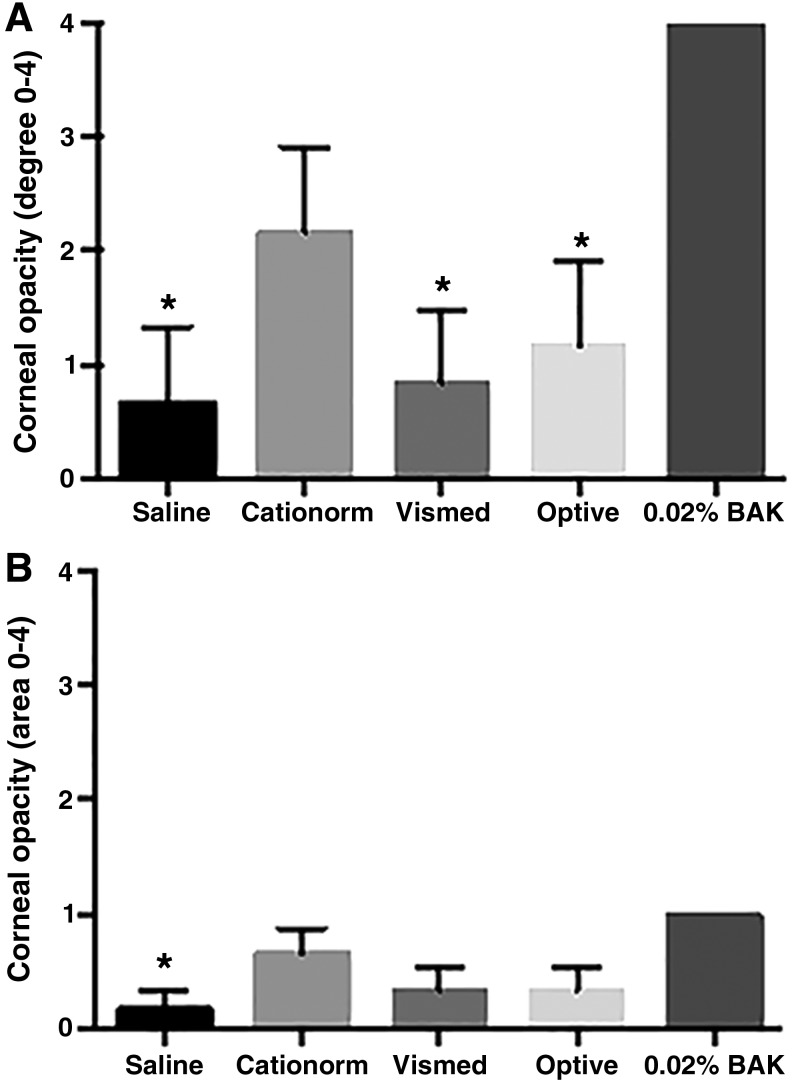
Mean McDonald–Shadduck's test score (±SEM) for the different treatment groups at day 3 for corneal opacity degree **(A)** and corneal opacity area **(B)**. *P < 0.05 (1-way ANOVA, Tukey's multiple comparison test) versus 0.02% BAK.

Examination of the ocular surface (cornea and conjunctiva), as well as the inner parts of the eye (iris) and the aqueous flare, was performed, with a slit lamp and by IVCM at baseline, during the treatment period, and at the end of the experiment to characterize the potential side effects that may result from the high number of daily instillations. Ocular examination of the cornea, conjunctiva, and iris before the first and after the 10th and 21st instillations of each day did not reveal any major findings throughout the experiment. The resulting mean score per group on the conjunctiva and cornea using the Draize's scale (Fig. 4) remains extremely low for all treatments, including the 0.02% BAK solution. The only findings (minor in intensity and duration) that affected the conjunctiva: slight conjunctival discharge and redness present in most of the eyes of each group. They were seen only on the first day of instillation just after the corneal abrasion. No findings were observed on days 2 and 3 (Fig. 4). Slit lamp biomicroscopic observations on day 3 revealed a slight conjunctiva congestion and a slight iris hyperemia for one eye in the Vismed group. The other findings affected the cornea. Some opacities were observed for one eye in the saline group, 2 eyes in the Vismed and Optive groups, 4 eyes in the Cationorm group, and for all the eyes in the 0.02% BAK group (Fig. 5). However, the overall mean score per group on the McDonald–Shadduck's scale remains low, even for the 0.02% BAK group. There was no statistically significant difference between saline and the 3 commercially available ATs (Cationorm, Vismed, and Optive); however, saline, Vismed, and Optive were significantly different from the 0.02% BAK.

**FIG. 6.**
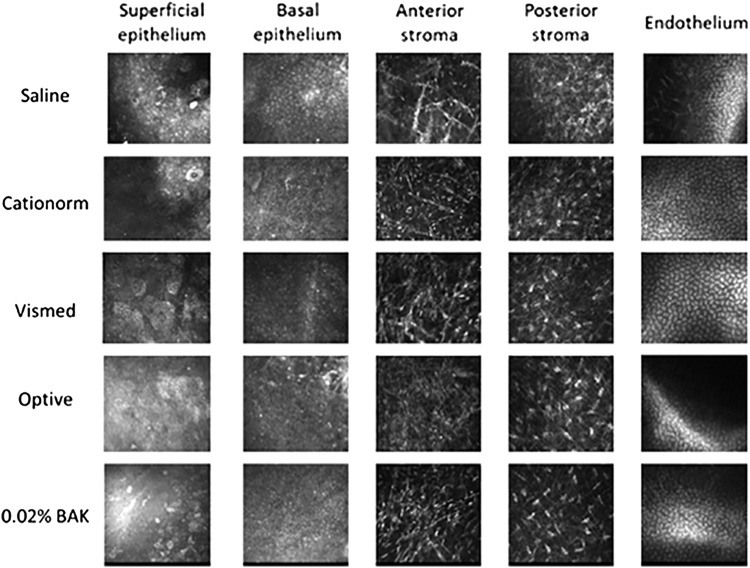
IVCM representative pictures of the superficial and basal epithelium, the anterior and posterior stroma, and the endothelium of the cornea for the different treatment groups. IVCM, *in vivo* confocal microscopy.

IVCM pictures of the superficial and basal epithelium, the anterior and posterior stroma, and the endothelium (Fig. 6) did not reveal significant differences between the AT groups and the saline group. Superficial epithelial cells appeared to be relatively large with an irregular size, with some smaller cells visible in the 0.02% BAK and saline groups. In a basal epithelium that looked overall homogeneous within the different groups, areas with irregular intercellular spaces were visible, with hyper-reflective cells being more present in the 0.02% BAK and Optive groups compared to the other treatments. The anterior stroma in the 0.02% BAK group presented numerous hyper-reflective elongated spindle-shaped cells with a linear cell body suggesting apoptosis. Fewer presumably apoptotic cells can also be seen in the anterior stroma from the other treatment groups. The posterior stroma and endothelium appeared to be normal for all treatment groups.

## Discussion

DED is a very common eye disease that affects millions of people worldwide.^24^ It manifests by symptoms of discomfort associated with clinical signs of corneal and conjunctival alterations. The vast majority of DED patients experience mild-to-moderate signs and symptoms that are generally treated by repeated instillations of ATs.^25^ While the patients are advised to instill ATs when necessary, that is, upon feeling sensations of discomfort, the dosage regimen of ATs for the alleviation of DED symptoms rarely exceeds 4 instillations per eye and per day.^13,26^ However, for the management of very severe symptoms, more frequent instillations of ATs might be needed on an acute basis (eg, one drop per hour) before switching to prescription (Rx) eye drops designed for the treatment of severe DED conditions.^18,19^ An hourly dosing regimen in a 12-h awaken period per day would represent 12 eye drops per day, quite a high burden for the patient. Indeed, an hourly dosing regimen is not sustainable for the patients in the long term, especially when Rx medicine containing potent (and safe) anti-inflammatory compounds, such as cyclosporine, designed for the treatment of severe DED conditions in dry eye patients that did not improve with ATs, are available and were demonstrated to be effective following the instillation of just one drop per day and per eye.^19,27,28^

Recently, an *ex vivo* eye irritation assay was used to compare commercially available ATs with the goal of exploring the toxicity of ATs.^29^ To make sure that some toxic events arise following the instillations of the ATs an hourly dosage regimen (ie, 24 eye drops a day) was applied over the 3-day experiment. The cultured half eye balls received 72 eye drops over a 3-day period. With such overdosing toxicity side effects were indeed observed in this EVEIT assay for Cationorm and Optive.^29^

The aim of the present work was to explore *in vivo* the potential side effects that may be induced on abraded rabbit cornea epithelium by Cationorm and other commercially available ATs following a high dosing regimen. Four small and standardized abrasions were performed on the corneas of rabbits' right eye before receiving one eye drop every 45 min, that is, 21 instillations per day over a 3-day period (except on the day of induction with 16 instillations). This design was chosen to mimic *in vivo* the extreme instillation protocol (24 eye drops per day) used in the *ex vivo* irritation test, EVEIT, and confirm, or not, the relevance of the EVEIT assay as an alternate toxicity test method for the evaluation of eye drops.

The data presented on Figs. 2 and 3 demonstrate that the healing process in the rabbit is normal and that even one instillation every 45 min (ie, 58 instillations over 3 days) of Cationorm for 3 days has no negative impact on abraded rabbit eyes compared to saline instillations. Indeed, BID instillations of cationic emulsions^30,31^ were demonstrated to contribute to the promotion of the healing process of abraded cornea in a rat scrapping assay. The IVCM pictures (Fig. 6) confirmed structurally that the corneal epithelium did heal properly. In addition, in an *in vivo* rabbit acute toxicity assay, the animals received 15 instillations over a 90-min period, that is, one instillation every 5 min for 90 min; no toxic reactions were found following the applications of cetalkonium chloride (CKC)-containing cationic emulsions.^32^ During the development process of the cationic emulsions (Cationorm, Ikervis), the 28-day local tolerability studies (6 daily instillations over 28 days) confirmed that the cationic emulsions, with CKC as the cationic agent, were very well tolerated by rabbit eyes.^33,34^ This good safety profile in animal models was also observed in patients during the different clinical trials evaluating the safety and efficacy of the cationic emulsions.^10,13,19,28^ For example, in the Nosika trial, Cationorm *vs.* Vismed, 4 instillations per day over 3 months in patients with mild-to-moderate DED signs and symptoms; 22.7% of the patients treated with Cationorm have a complete cornea clearing (ie, a completely healed cornea) as soon as 7 days after treatment initiation.^13^ By comparison, Vismed treatment reached this effectiveness (22.0%) only after 3 months of treatment.^13^ In addition, the annual update safety reports of Cationorm also confirmed that Cationorm is very well tolerated by the altered and inflamed ocular surface of DED patients.

The improved wound healing and anti-inflammatory properties of the cationic emulsions demonstrated during clinical trials (Nosika and Sansika trials) in patients were suggested previously by *in vitro* studies in human corneal epithelial cells and *in vivo.*^30,31,35^ Indeed, in these early experiments, it was noticed that the cationic emulsions were able to improve the wound healing process (both in pace and “quality”). The reason for the wound healing clinical efficacy of the cationic emulsions was further explored at the mechanistic level. Recent studies demonstrated that CKC was a specific inhibitor of protein kinase C (PKC) alpha^36^ (Daull, 2018, in press). Recently, Pflugfelder and collaborators (2010) demonstrated that PKCα “inhibition” improved wound healing by reducing the level of inflammatory cell infiltration in the wounded area with PKCα knockout mice which have a “*more rapid corneal epithelial wound healing, perhaps due to a decreased neutrophil infiltration.*”^37^ Hence, the inhibition of PKCα by CKC might explain why cationic emulsions like Cationorm and the Ikervis vehicle did perform so well in the improvement of corneal epithelium healing in the clinical trials.^13,19^

Following the rationale for reducing the use of live animals in toxicology studies, alternate toxicity test methods are being developed. These new *in vitro* (and *ex vivo*) test methods are particularly valuable for the scientific community and the society in general. However, these alternate test methods used for toxicity evaluation need an extended validation phase to make sure that they produce reliable data that can confidently be used to adequately assess the toxicity of the tested eye drops or substances.^38^ Unfortunately, most of these alternate irritation assays failed in their validation phase. Indeed, the interagency coordinating committee on the validation of alternative methods (ICCVAM) and ICCVAM Ocular Toxicology Working Group did not recommend the use of the bovine corneal opacity and permeability, Cytosensor^®^ Microphysiometer (CM), hen's egg test-chorioallantoic membrane, isolated chicken eye, and isolated rabbit eye test methods to distinguish substances not labeled as irritants.^23^ In most of the cases the false positive rates were high. Recently, Frentz et al. developed an EVEIT as an alternative to the *in vivo* evaluation of toxicity or ocular tolerability.^39,40^ So far, the EVEIT assay has not yet undergone formal validation^41^ and was not even listed as an alternate irritation test method by Abdelkader et al. in 2015^42^ and Lotz et al. in 2016^43^ in their reviews.

In a direct comparison of the EVEIT assay and an *in vivo* assay in rabbits (this study) based on the Draize test, the most reliable eye irritation test, used for more than 70 years^44^ in drug development to address regulatory requirements for safety testing, it appears that the EVEIT assay failed to adequately predict the toxicity of compounds not classified as irritant. In conclusion, the EVEIT as it is presently designed and used appears not to be an adequate alternate test method for safety testing of eye drops. Modifying its design so that the EVEIT test method more closely mimics the eye's normal lubrication and physiology would probably improve its predictability and decrease its overprediction rate and position this test method, once it has passed formal validation, as a potential alternate toxicity assay for eye drops.
